# Fluorescence Lifetime Imaging of NAD(P)H in Patients’ Lymphocytes: Evaluation of Efficacy of Immunotherapy

**DOI:** 10.3390/cells14020097

**Published:** 2025-01-10

**Authors:** Diana V. Yuzhakova, Daria A. Sachkova, Anna V. Izosimova, Konstantin S. Yashin, Gaukhar M. Yusubalieva, Vladimir P. Baklaushev, Artem M. Mozherov, Vladislav I. Shcheslavskiy, Marina V. Shirmanova

**Affiliations:** 1 Institute of Experimental Oncology and Biomedical Technologies, Privolzhsky Research Medical University, 10/1 Minin and Pozharsky Sq., 603005 Nizhny Novgorod, Russia; sachkova.collins@gmail.com (D.A.S.); annizosimova@mail.ru (A.V.I.); artemmozherov@gmail.com (A.M.M.); shirmanovam@gmail.com (M.V.S.); 2 Institute of Biology and Biomedicine, Lobachevsky State University of Nizhny Novgorod, 23 Gagarin Ave., 603950 Nizhny Novgorod, Russia; 3 Department of Neurosurgery, Privolzsky Research Medical University, 10/1 Minin and Pozharsky Sq., 603005 Nizhny Novgorod, Russia; jashinmed@gmail.com; 4 Federal Research and Clinical Center, Federal Medical and Biological Agency, 28 Orekhovy Blvd., 115682 Moscow, Russia; kakonya@gmail.com (G.M.Y.); baklaushev@fccps.ru (V.P.B.); 5 Laboratory of Molecular Mechanisms of Regeneration and Aging, Engelhardt Institute of Molecular Biology, Russian Academy of Sciences, 32 Vavilova St., 119991 Moscow, Russia; 6 R&D Department, Becker&Hickl GmbH, 7-9 Nunsdorfer Ring, 12277 Berlin, Germany

**Keywords:** immunotherapy, immune-checkpoint inhibitor ICI, personalized therapy, tumor explant, lymphocyte, glioma, fluorescence lifetime imaging FLIM, metabolism, NAD(P)H

## Abstract

Background: The wide variability in clinical responses to anti-tumor immunotherapy drives the search for personalized strategies. One of the promising approaches is drug screening using patient-derived models composed of tumor and immune cells. In this regard, the selection of an appropriate in vitro model and the choice of cellular response assay are critical for reliable predictions. Fluorescence lifetime imaging microscopy (FLIM) is a powerful, non-destructive tool that enables direct monitoring of cellular metabolism on a label-free basis with a potential to resolve metabolic rearrangements in immune cells associated with their reactivity. Objective: The aim of the study was to develop a patient-derived glioma explant model enriched by autologous peripheral lymphocytes and explore FLIM of the redox-cofactor NAD(P)H in living lymphocytes to measure the responses of the model to immune checkpoint inhibitors. Methods: The light microscopy, FLIM of NAD(P)H and flow cytometry were used. Results: The results demonstrate that the responsive models displayed a significant increase in the free NAD(P)H fraction α_1_ after treatment, associated with a shift towards glycolysis due to lymphocyte activation. The non-responsive models exhibited no alterations or a decrease in the NAD(P)H α_1_ after treatment. The FLIM data correlated well with the standard assays of immunotherapy drug response in vitro, including morphological changes, the T-cells activation marker CD69, and the tumor cell proliferation index Ki67. Conclusions: The proposed platform that includes tumor explants co-cultured with lymphocytes and the NAD(P)H FLIM assay represents a promising solution for the patient-specific immunotherapeutic drug screening.

## 1. Introduction

In recent decades, immunotherapy has developed significantly to allow complete and long-lasting tumor regression for different types of malignancies including melanoma, non-small cell lung, bladder cancer, and renal cell carcinoma, leukemia, and lymphoma [1]. With respect to some other types of tumors, e.g., multiple myeloma, hepatocellular carcinoma, and gliomas, immunotherapy is undergoing clinical trials and demonstrates promising results [2]. Among different strategies used to activate the immune system against tumor cells, the immune-checkpoint inhibitors, such as anti-programmed death receptor-1/programmed cell death receptor-ligand-1 (anti-PD-1/PD-L1) and anti-cytotoxic T lymphocyte associated antigen-4 (anti-CTLA-4) monoclonal antibodies are widely recognized [3,4].

However, despite its remarkable efficacy, treatment resistance and toxicity are serious limitations of immunotherapy. The response to immunotherapy significantly varies among patients with the same tumor type, so that only a small subset of patients respond to the therapy. Other patients either do not respond at the onset of treatment (primary resistance) or develop resistance following the initial response, leading to relapse (acquired or secondary resistance). Resistance to immunotherapy is a complex and multifactorial phenomenon, the underlying mechanisms of which are not yet fully understood. It is considered that the resistance to immunotherapy can be associated with extrinsic factors, i.e., related to the tumor microenvironment, and intrinsic factors, i.e., related to tumor cells [5]. The identification of patients who will respond to the treatment and benefit from it is one of the biggest challenges in the field of immunotherapy.

Personalized prediction of immunotherapy outcomes is currently developing in two main directions. The first is associated with the search for effective biomarkers of clinical efficacy. A limited number of useful biomarkers exist that predict immunotherapy response; they are the PD-L1 expression level and tumor mutational burden [6]. As potential biomarkers, the features of tumor tissue, tumor microenvironment, peripheral blood components, and microbiome are considered. An increasing number of biomarker candidates are being proposed on the basis of genomic and transcriptomic data [7,8]. However, the prediction of responses using a single biomarker is difficult due to the complexity of the tumor and the human immune system. Multiparametric analysis, which may include tumor genomics, immune gene signatures, immunohistochemistry, and/or a blood-based assay, could be more informative, but its introduction into routine clinical practice seems unrealistic.

An alternative approach to personalized immunotherapy is drug screening on patient-specific cellular models containing patient-derived tumorous and immune cells [9,10]. Creating an experimental model that recapitulates the interaction between the tumor, the immune system and the host is a challenging task. An ideal model should, on the one hand, closely resemble the biological features of patients’ tumors and tumor microenvironment, and, on the other hand, be highly reproducible, easy to maintain and provide an immediate and reliable response to the treatment. Different in vitro platforms have been proposed so far for immunotherapy assessment, including 2D co-cultures of tumor cells and immune cells and 3D structures, like spheroids, organoids, tissue explants, bioprinted scaffolds, and tumor-on-a-chip microfluidic systems [11,12]. Despite the variety of models available, there is still no standard that would maintain the right balance between “close-to-patient” and “easy-to-use”. Another challenge in the development of an in vitro platform for the prediction of immunotherapy efficacy is the choice of endpoint analysis method. For this, different viability and proliferation assays on cancer cells and activation markers assays on immune cells are traditionally used, which often require the dissociation of the 3D structure into a single cell suspension, fixation and staining and, therefore, make it impossible to repeat measurements on the same object and observe cells in their natural state.

Recent studies have reported the use of fluorescence lifetime imaging microscopy (FLIM-microscopy, further simply FLIM) for the identification of the drug response of cancer cells and the activation of immune cells [13] by registration of cellular decay profiles of autofluorescence. Specifically, fluorescence lifetime of the redox cofactor NAD(P)H varies depending on the protein binding and take on short (0.4 ns) or long (1.5–4.5 ns) values for the free and bound states, correspondingly [14]. Therefore, any cellular processes mediated by the changes in NADH- and NADPH-dependent metabolic pathways (e.g., glycolysis, the TCA cycle, mitochondrial respiration, β-oxidation of fatty acids, redox balance), can be assessed with this technique. Owing to its non-invasiveness, high sensitivity, label-free principle, and quantitative readouts, FLIM has become a powerful technique for in vitro and in vivo estimation of cellular metabolism on the microscopic scale [15,16]. However, the potential of this method for personalized prediction of immunotherapy efficacy is largely unexplored.

The purpose of this study is to develop a platform for personalized immunotherapeutic drug screening using the co-culture of patient-derived tumor explants and autologous lymphocytes and FLIM of NAD(P)H as the end-point analysis. The explants culture model was selected for the study because tumor fragments preserve all tissue components, thus retaining the histological features and immune microenvironment of the original tumors [17]. Patient-derived explant cultures are easy to handle and can be obtained for various tumor types, including those for which primary cell culture cannot be established. The performance of the platform was validated using the explant cultures derived from patients’ gliomas. Cellular metabolic changes in lymphocytes co-cultured with glioma explants were assessed by FLIM in response to immunotherapy with anti-PD-1/PD-L1 and anti-CTLA-4 antibodies and their combination. The activation of T-lymphocytes and the inhibition of glioma cell proliferation were verified by flow cytometry and biochemical methods.

## 2. Materials and Methods

### 2.1. Patient Samples

All studies on patients’ material were approved by the Local Ethical Committee of PRMU (protocol #12 from 5 August 2022). Informed signed consents were obtained from the participants prior to inclusion in the study. Tumor and peripheral blood samples were obtained from 14 patients of the University Clinic, PRMU, with diagnosed glioma.

Freshly resected samples of the tumors, 7–10 mm in size, were transported from the clinic to the laboratory within 1 h in 50 mL falcons with DMEM (Dulbecco’s modified eagle medium)/F12 medium with a 2-fold concentration of antibiotic–antimycotic (Gibco, Amarillo, TX, USA) on ice. On the same day, 10 mL of peripheral blood was drawn from each patient into vacuum blood collection tubes containing EDTA (ethylenediaminetetraacetic acid) and transported on ice.

The dataset comprised tumor and blood samples from fourteen individuals (six males, eight females) aged 28 to 73. Among the tumor samples there were 6 glioblastomas (WHO grade 4), 5 astrocytomas (WHO grade 2–4), 2 oligodendrogliomas (WHO grade 3), and 1 oligoastrocytoma (WHO grade 3). Six of the fourteen tumors had the wild-type IDH-status and the other eight had mutations in the IDH1/IDH2 genes. Twelve of fourteen tumors were primary without any previous treatment, while the remaining two patients with a recurrent tumor received the surgical resection alone or with radiotherapy and chemotherapy with temozolomide. It should be noted that the patients did not receive the immunotherapy either before or after surgery.

The information about the samples is presented in Table 1.

### 2.2. Experimental Design

**Scheme 1:**
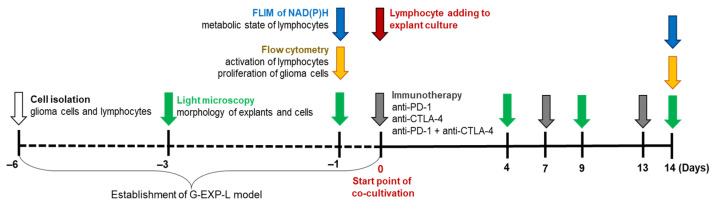
The experimental workflow.

The experimental workflow is demonstrated in Scheme 1. At the first stage (from day −6 to day 0), patient-derived explants of glioma and, in parallel, lymphocyte cultures were established and characterized by light microscopy, FLIM, and flow cytometry. On day 0, lymphocytes were added to glioma explants simultaneously with immunotherapeutic agents. At the second stage (from day 0 to day 14), the resulting patient-specific G-EXP-L (Glioma-EXPlant-Lymphocytes) models were treated on days 7 and 13. Morphological phase contrast images of the treated and untreated control G-EXP-L models were obtained on days 4, 9, and 14 of co-culturing. Flow cytometry and FLIM were performed 14 days after the start of treatment.

Routine observations of cell morphology were performed on the inverted light microscope Leica DM IL LED (Leica, Wetzlar, Germany). Phase contrast images were obtained at the 10× and 40× magnifications.

### 2.3. Patient-Derived Explants of Glioma

The protocol for the preparation of tumor explants was adopted from our previous paper [18]. Briefly, explants cultures were obtained from fresh tumor specimens, which were dissected with a scalpel into small fragments that were then cultured in an RPMI-1640 medium in a CO_2_ incubator. The detailed procedure is described in the Supplementary Materials.

The characteristics of the resulting patient-derived glioma explant cultures on day 1 is presented in Supplementary Table S1. On day 0, when the immune cells and immunotherapeutic agents were added to the explants, the tumor cell confluence reached approximately 30% (Supplementary Figure S1).

### 2.4. Lymphocyte Isolation and Culturing

The lymphocyte isolation was performed using the Histopaque-1077 (Sigma-Aldrich, Stockholm, Sweden) according to the manufacturer’s protocol. Lymphocytes were cultured in an RPMI-1640 medium with L-glutamine and HEPES with the addition of 0.1% human interleukin 2, 10% FBS, and 0.1% penicillin–streptomycin. Details of the procedure of isolation and culturing can be found in the Supplementary Materials.

Lymphocytes were added to autologous explant culture at a concentration of 2 × 10^5^ cells per well. The resulting patient-specific G-EXP-L models were cultured without passing over 2 weeks. If necessary, one third of the medium (600 μL per well) was replaced with fresh one.

### 2.5. Immunotherapy

Anti-PD-1 Nivolumab (Opdivo) and anti-CTLA-4 Ipilimumab (Yevroy) (Bristol-Myers Squibb, Princeton, NJ, USA) antibodies were used as monotherapy or in a combination. Anti-PD-1 and anti-CTLA-4 antibodies were added to the G-EXP-L model at the final concentration of 5 μg/mL [19] on days 0, 7, and 13 of co-cultivation. For each patient there were 2–3 control wells with the untreated G-EXP-L model and 2–3 wells with each type of treatment.

### 2.6. FLIM of NAD(P)H in Lymphocytes

The medium containing the immune cells was removed from each well, divided into two parts for analysis by FLIM and flow cytometry, respectively, and centrifuged at 300× *g* for 5 min. For visualization, the immune cells were resuspended in 50 µL of FluoroLite™ DMEM without phenol red (Gibco, Amarillo, TX, USA), placed in 96-well black/clear plate (Falcon^®^, Corning Incorporated, Berlin, Germany) and incubated for 5–10 min in a CO_2_-incubator for cell sedimentation.

The detailed protocol of FLIM of NAD(P)H is described elsewhere [18,20]. Briefly, the imaging was performed using a laser scanning microscope Zeiss LSM 880 (Carl Zeiss, Jena, Germany) equipped with a fs laser (Spectra-Physics, Milpitas, CA, USA) and a single photon counting card SPC-150N, (Becker&Hickl GmbH, Berlin, Germany) with a hybrid detector HPM-100-40 (Becker&Hickl GmbH, Berlin, Germany). The excitation of NAD(P)H was performed at 750 nm with a detection of fluorescence in the spectral range from 450 to 490 nm.

### 2.7. Flow Cytometry

BD FACSAria III cell sorter (BD Biosciences, San Jose, CA, USA) and FlowJo v10.5.3 software (BD/Treestar, Ashland, OR, USA) were used for analyzing and data processing, respectively.

The protocols of flow cytometry of lymphocytes for analysis of their activation and glioma cells for evaluation of their proliferative index were adopted from our previous papers Izosimova et al. [20] and Yuzhakova et al. [18], respectively, and are presented in the Supplementary Materials.

### 2.8. Statistical Analysis

The protocol of the statistical analysis including the software and the test for the calculation of the normality of distribution was adopted from our previous paper Yuzhakova et al. [18]. Data are presented as means ± SD or means ± SEM; statistical significance was evaluated using paired or unpaired Student’s *t*-test or Mann–Whitney U test (*p*-value ≤ 0.05 was considered statistically significant), as indicated in the respective figure legends.

## 3. Results

### 3.1. Characterization of G-EXP-L Model

*Cell morphology.* Patient-derived G-EXP-L model consisted of 3–4 adherent tumor fragments per well, surrounded by radially migrating adherent tumor cells and non-adherent lymphocytes located above them.

**Figure 1:**
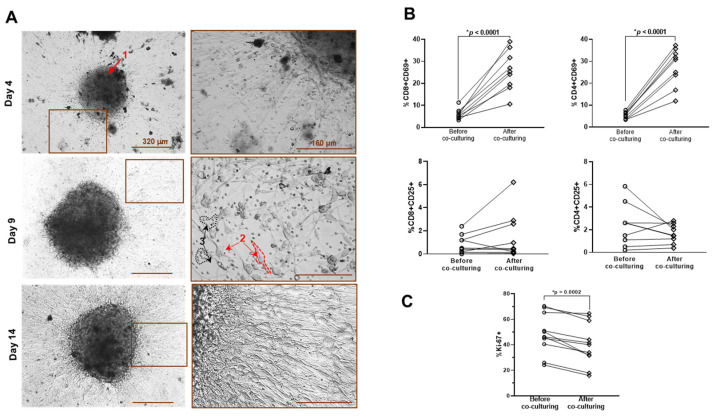
Characteristics of G-EXP-L model. (**A**) Phase contrast microscopy of patient-derived G-EXP-L model on days 4, 9, and 14 of cultivation. The components of the model are shown by the numerated arrows: 1—adherent tumor fragment, 2—adherent tumor cells, 3—lymphocytes. (**B**) Expression of the activation markers CD69 and CD25 in live CD4+ and CD8+ T-cells before and after co-culturing with glioma explants. Dot plots show the measurements for the individual patients (dots), and horizontal lines connect the values for the same patient before and on day 14 of co-culturing. (**C**) Expression of proliferation marker Ki67 in tumor explants before and on day 14 of co-culturing. Dot plots show the measurements for individual patients (dots), and horizontal lines connect the values for the same patient before and after co-culturing. Statistics: paired Student’s *t*-test. * Significant difference, *p* ≤ 0.05.

Adherent tumor fragments (explant) had a round, oval, or irregular shape and inhomogeneous structure with a light periphery consisting predominantly of living proliferating cells and a dark inner core containing a number of dead cells (Figure 1A). During 14 days of culturing, most tumor fragments increased in size (from 100 to 350 μm in diameter on day 4 to a size of 400–600 μm in diameter on day 14), and their structure became denser—the dark central core with necrotic areas increased, while the layer of proliferating cells at the periphery was preserved.

Single adherent tumor cells were generally diffusely distributed in the wells, thus forming a monolayer, with a tendency to form radial “rays” around the tissue fragments. These cells displayed moderate intratumoral and pronounced intertumoral polymorphism. There were the cells of different morphology—spindle-shaped, oval, round, triangular, or elongated large cells marked by cytoplasmic granularity and numerous prolongations. As glioma cells proliferated in a culture, their confluence increased to 80% on the 14th day of G-EXP-L model cultivation, compared to 30% on the 4th day.

Lymphocytes demonstrated non-uniform distribution with a tendency to form clusters around tumor cells. When co-cultured with glioma cells, they showed morphological changes typical of activated immune cells, such as an increase in size and/or elongation [21,22]. During the cultivation of G-EXP-L, the number of immune cells did not change significantly. 

*T-cells activation.* Co-cultivation of lymphocytes with glioma explants affected the expression of CD69, an early marker of lymphocyte activation. Percentage of CD69+ cells increased in both CD8+ (*p* = 0.0116) and CD4+ T-cell subsets (*p* = 0.0097) (Figure 1B). The expression of the CD25+ activation marker demonstrated the heterogeneous tendency in different patient-derived models. The models G20, G22, G32, and G37 demonstrated the increase in the percentage of CD69+ cells in the CD8+ T-cell subset, while the rest of the models showed no pronounced changes. Concerning the CD4+ T-cell subset, the models G20, G32, and G37 demonstrated an increase in the percentage of CD69+ cells, while the rest of the models demonstrated no changes (G22 and G29) or decreases (G27, G30, and G31) in the percentage of CD69+ cells. Changes in the CD4+CD25+ T-cell–cell subset may be due to changes in the percentage of activated effector CD4+ T helper cells or the percentage of immunosuppressive regulatory CD4+FoxP3+ T cells.

*Ki67 in tumor cells.* Glioma cells from different patients initially demonstrated a high interpatient variability of proliferative index Ki67, from 24% to 70%. Co-cultivation with lymphocytes resulted in a statistical decrease in the percentage of Ki67+ glioma cells (*p* = 0.009) in all G-EXP-L models to the value from 16% to 65% (Figure 1C). A decreased proliferative activity of tumor cells in the presence of lymphocytes may be associated with the suppressive effect of immune cells [23,24].

**Figure 2:**
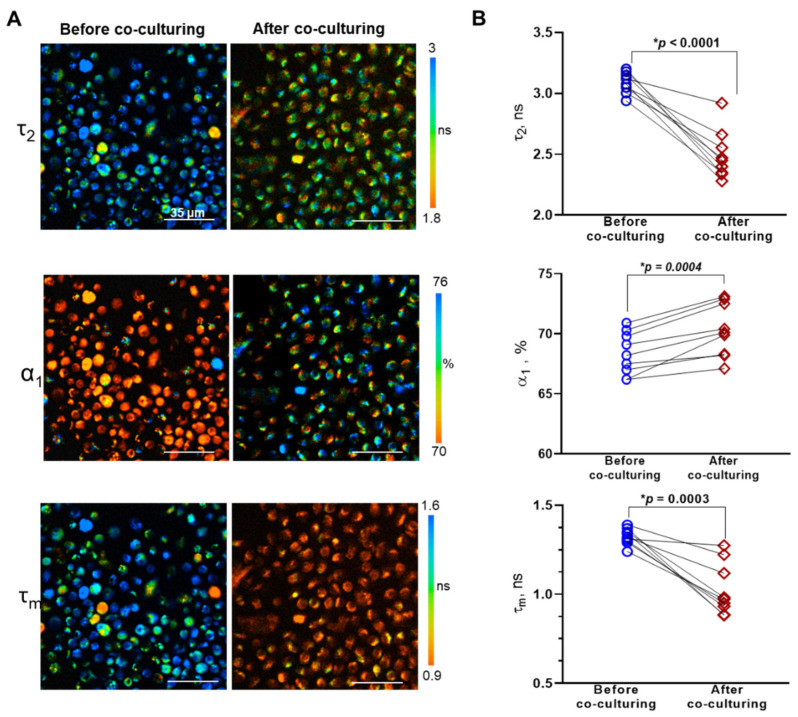
FLIM of NAD(P)H in lymphocytes before and after co-culturing with glioma explants. (**A**) Representative FLIM images of lymphocytes before and after co-culturing. The long lifetime component of NAD(P)H τ_2_, the relative contribution of free NAD(P)H α_1_, and the mean lifetime τ_m_ are shown. (**B**) Coupled comparisons of fluorescence decay parameters (τ_2_, α_1_, τ_m_) before and after co-culturing. Dots are the measurements for individual patients. Coupled comparisons of NAD(P)H-α_1_ before and after co-culturing. Dots are the mean values for individual patients. Lines connect the values before and after co-culturing for the same patient. Statistics: paired Student’s *t*-test. * Significant difference, *p* ≤ 0.05.

*NAD(P)H fluorescence lifetimes in lymphocytes.* Autofluorescence lifetime parameters of NAD(P)H were analyzed in the lymphocytes of each patient in 14 days after their cultivation in the G-EXP-L model and compared with those in naive lymphocytes from the same patients (Figure 2).

In lymphocytes before co-cultivation with glioma explants, the short lifetime value, corresponding to free NAD(P)H, τ_1_ was 0.5 ± 0.01 ns on average. The long lifetime τ_2_, corresponding to protein-bound NAD(P)H, in the individual samples varied from 2.94 ± 0.02 to 3.2 ± 0.07 ns, the mean lifetime τ_m_ varied from 1.24 ± 0.04 to 1.35 ± 0.05 ns (Supplementary Table S2). Relative contribution of free NAD(P)H α_1_ was in the range of 66.2 ± 0.5% to 70.9 ± 1.3%.

Lymphocytes in all the G-EXP-L models had shorter fluorescence lifetime values compared with corresponding naive cells—τ_1_ 0.4 ± 0.01 ns on average (*p* = 0.0001), τ_2_ from 2.28 ± 0.03 to 2.92 ± 0.07 ns (*p* < 0.0001), τ_m_ from 0.88 ± 0.01 to 1.27 ± 0.04 ns (*p* = 0.0003). The fraction of free NAD(P)H α_1_ increased in all models and was in the range of 67.1 ± 0.6 to 73.1 ± 0.5% (*p* = 0.0004). The observed changes in fluorescence decay parameters are most likely associated with a modified profile of NAD(P)H-binding enzymes (τ_2_) and a glycolytic shift (α_1_), the last of which is known to accompany the activation of T-cells in the presence of tumor cells [20,25,26].

To validate the sensitivity of NAD(P)H FLIM to perturbations targeting energy metabolism of T-lymphocytes, the experiments on naïve T-cells from healthy donors treated with standard metabolic inhibitors 3-bromopyruvate (3-BP) and rotenone were performed (Supplementary Figure S2). It was shown that NAD(P)H α_1_ statistically decreased upon inhibition of glycolysis with 3-BP and increased upon inhibition of oxidative phosphorylation with rotenone. At that NAD(P)H τ_2_ value did not statistically change. As a result, the mean lifetime value τ_m_ increased after treatment 3-BP and decreased after treatment with rotenone. This suggests that changes in NAD(P)H fluorescence decay parameters observed in T-cells after co-culturing with glioma explant culture could be attributed to the increased glycolytic rate.

Therefore, we developed a 3D model G-EXP-L based on fresh tumor explants from individual glioma patients and autologous peripheral lymphocytes. In this model a cytotoxic effect of immune cells on glioma cells was detected, as reflected by the activation of T cells in the presence of tumor antigens and a decrease in the proliferative index of glioma cells. The cross-talk between lymphocytes and glioma cells resulted in metabolic rearrangements in the lymphocytes with a shift to a more glycolytic state, as assessed from NAD(P)H FLIM data.

### 3.2. Effects of the Therapy by Immune Checkpoint Inhibitors on T Lymphocytes in the G-EXP-L Model

The G-EXP-L models were obtained from the material of 14 patients—each was treated with one to three immunotherapy regimens (anti-CTLA-4, anti-PD-1 or combination), depending on the amount of available resection material. In total, 28 tests (patient/regimen) were implemented.

#### 3.2.1. Morphological Changes in the G-EXP-L Model After Treatment

**Figure 3:**
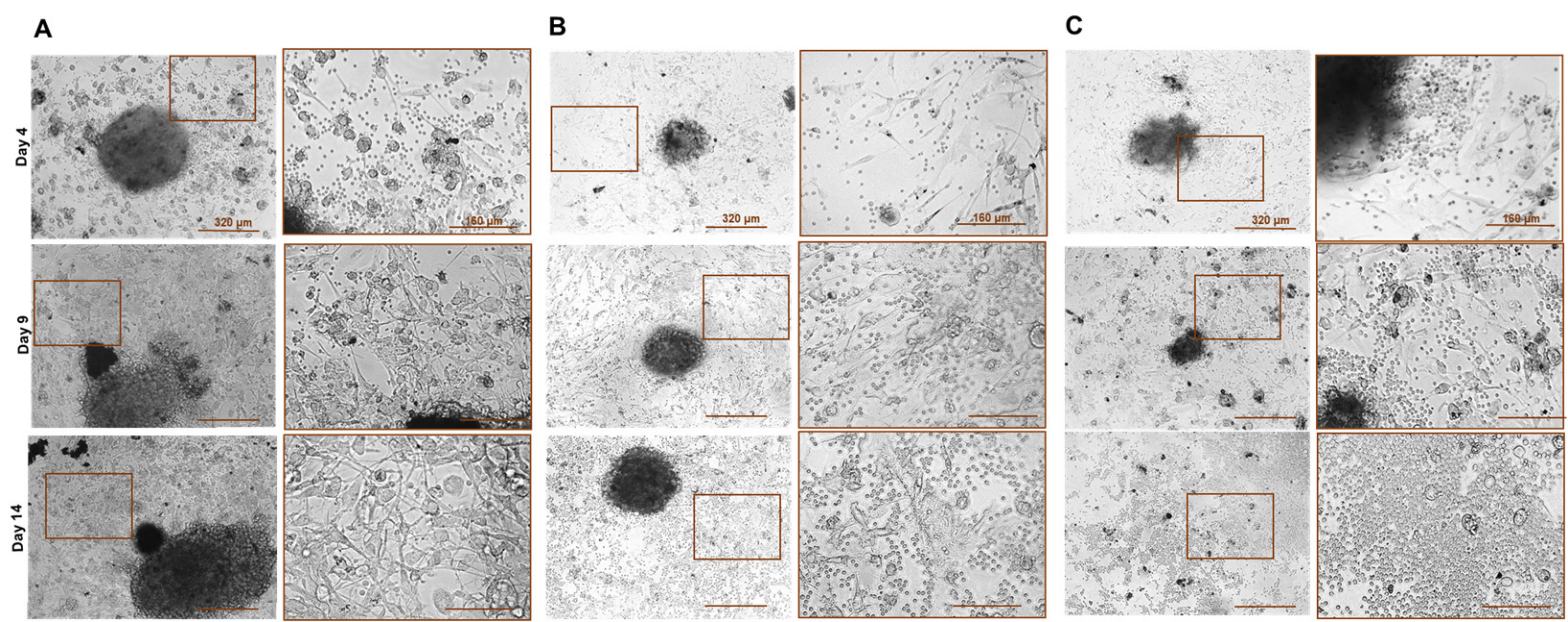
Phase contrast microscopy of patient-derived G-EXP-L model after treatment with immune checkpoint inhibitors on the days 4, 9, and 14 of cultivation. Examples of the morphological response: (**A**)—“no response” (patient G27, anti-CTLA-4 treatment), (**B**)—“partial response” (patient G20, anti-PD-1 treatment), (**C**)—“response” (patient G30, combination) treatment. Bars are indicated in the images.

To evaluate the effects of the therapy on G-EXP-L models, morphological features of all components of the model—adherent tumor fragment, tumor cell monolayer and non-adherent immune cells, were defined. Depending on the morphological features, all cases were classified as “no response”, “response”, and “partial response” (Figure 3).

G-EXP-L models in the group of “no response” were generally similar in their morphology to the untreated control of the same patient. Tumor fragments increased the size and density of the structure, and peripheral tumor cells formed a confluent monolayer (70–80% confluence). In the immune cell fraction, there was a significant decrease in the number of lymphocytes. Morphological “non-responses” to anti-CTLA-4 treatment were G23, G24, G27, and G33, to anti-PD-1—G27, G31, G32, G33, and G37; and to their combination were G27, G29, and G33.

In the case of complete response (“response” group), tumor explants gradually degraded, reducing in size and dissociating into small necrotic fragments by day 14. The number of tumor cells in a peripheral monolayer did not change or slightly increased by day 9, but then these cells were completely eliminated. The immune cell fraction significantly increased. Morphological “responses” to anti-CTLA-4 treatment were G16, G17, G20, G26, G30, and G37, to anti-PD-1—G17, G26, and G30; and to their combination were G20, G26, G30, and G37.

In the group of “partial response”, there was an inhibition of explant growth compared with the corresponding control from the same patient. The size of the explants either did not change or slightly increased. Tumor cell confluence in the peripheral monolayer did not exceed 50%. In the immune cell fraction, the number of lymphocytes significantly increased. Morphological “partial responses” to anti-CTLA-4 treatment were G22 and to anti-PD-1 treatment, G20 and G29.

Among all tests, complete response was observed in 13 of 28 cases, partial response in 3 cases, and no response in 12 cases.

#### 3.2.2. T-Cell Activation in the G-EXP-L Model After the Treatment

To verify the response of lymphocytes in the patient-derived models to immunotherapy, we assessed the expression of early response marker CD69 in the main effector T-cell subsets—CD8+ and CD4+.

**Figure 4:**
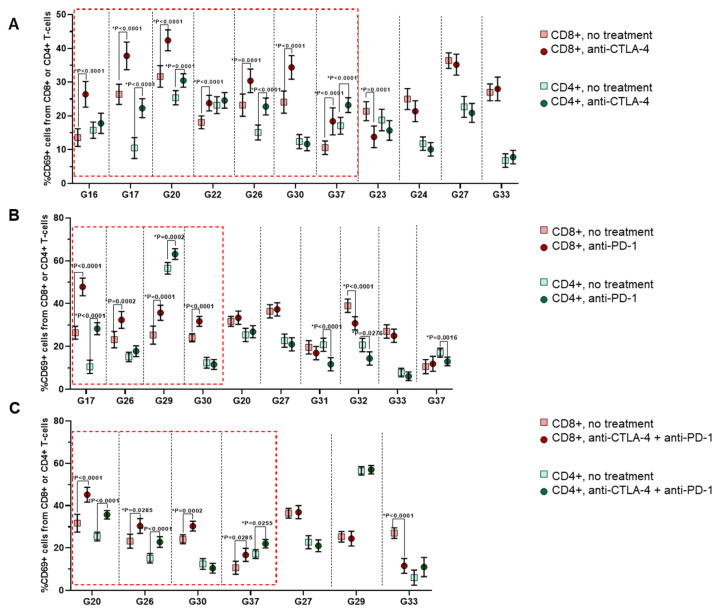
Expression of the activation marker CD69 in CD8+ or CD4+ T-lymphocytes in the G-EXP-L models after immunotherapy with anti-CTLA-4 (**A**), anti-PD-1 (**B**), or their combination (**C**). Dot plots show measurements for individual patients (dots) and SEM (horizontal lines). The red boxes indicate the cases of a significant rise in the percentage of CD69+ cells, either in the CD8+ T-cell subset or both CD8+ and CD4+ T-cells. Statistics: Mann–Whitney U test. * Significant difference, *p* ≤ 0.05.

After treatment with the anti-CTLA-4 antibodies, all morphological “responders” (G16, G17, G20, G26, G30, and G37) and “partial responders” (G22) demonstrated a significant increase in the percentage of CD69+ cells in CD8+ T-cell subset (*p* < 0.0001) in comparison with the untreated controls. Moreover, G17, G20, G26, and G37 models showed a statistically significant increase in CD69+ cells also in CD4+ T-cell subset (*p* ≤ 0.0001). In “non-responders”, there was no significant change in the percentage of CD69+ cells in both T-cell subsets (G24, G27, and G33) or that decreased in CD8+ T-cell subset (G23, *p* = 0.0001) (Figure 4A). 

A similar situation was observed with anti-PD-1 and combination therapies. Four “responders” to the anti-PD-1 (G17, G26, G29, and G30) had increased percentages of CD69+ cells in the CD8+ cell subset (*p* ≤ 0.0002), and two of them (G17 and G29)—also in CD4+ cells (*p* ≤ 0.0002). The “partial responder” (G20) showed no significant changes in the percentage of CD69+ cells either in CD8+ or in CD4+ T-cells. In “non-responders”, the CD69+ fraction either did not change (G27 and G33) or decreased in one (G37, *p* = 0.0016 and G31, *p* < 0.0001) or both T-cell subsets (G32, *p* ≤ 0.0276) (Figure 4B).

In the case of combination therapy, morphological “responders” G20, G26, and G37 had a higher percentage of CD69+ cells in both CD4+ (*p* ≤ 0.0255) and CD8+ (*p* ≤ 0.0285) T-cells subsets, and G30 only in the CD8+ subset (*p* = 0.0285) (Figure 4C). “Non-responders” G27 and G29 demonstrated no significant changes in the percentage of CD69+ cells either in CD8+ or CD4+ T-cells, while in the G33, the percentage of CD69+CD8+ T-cells decreased (*p* ≤ 0.0001).

Therefore, as anticipated, response to immunotherapy was accompanied by the rise in the percentage of activated CD69+ effector T-cells, predominantly within the CD8+ T-cell subset.

#### 3.2.3. Proliferative Index Ki67 of Glioma Cells in the G-EXP-L Model After Treatment

In addition to the effects occurring in the immune cells, we have also examined the effects of immunotherapy on tumor cells in the G-EXP-L models.

**Figure 5:**
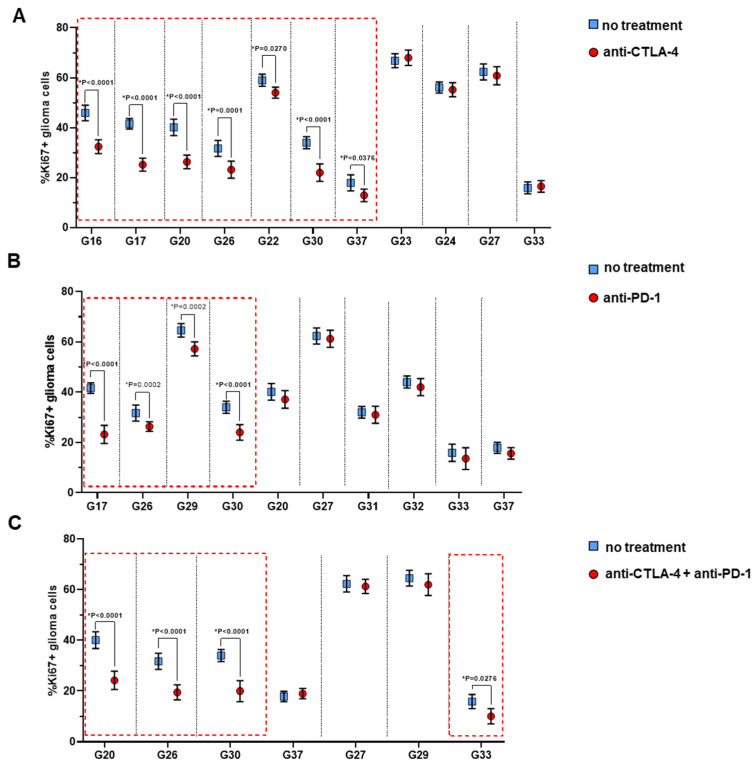
Expression of proliferative index Ki67 of glioma cells in the G-EXP-L models after immunotherapy with anti-CTLA-4 (**A**), anti-PD-1 (**B**) or their combination (**C**). Dot plots show measurements for individual patients (dots) and SEM (horizontal lines). The red boxes indicate the cases of a significant decrease in the percentage of Ki67+ glioma cells. Statistics: Mann–Whitney U test. * Significant difference, *p* ≤ 0.05.

With the anti-CTLA-4 and the anti-PD-1 treatment, all the “responsive” and “partially responsive” models demonstrated statistical decrease in the percentage of Ki67+ glioma cells in comparison with corresponding untreated controls (Figure 5). In all “non-responders” Ki67 proliferative index did not change after the treatment.

Combination therapy resulted in a decrease in the percentage of Ki67+ glioma cells in 3 of 4 “responsive” models, G20, G26, and G30 (*p* < 0.0001), and in 1 of 12 “non-responsive” models, G33 (*p* = 0.0276), while for other models no significant changes in the percentage of Ki67+ cells were observed. Therefore, in one model (G29) combination therapy was less effective than anti-PD-1 alone. The reasons for the lower efficacy of a combination are unknown; we can only speculate that combination therapy could provoke the expression of different immune checkpoints, which resulted in treatment resistance [27].

#### 3.2.4. FLIM of NAD(P)H in Lymphocytes After the Treatment

The autofluorescence decay parameters of NAD(P)H were analyzed in the lymphocytes from the G-EXP-L models after anti-CTLA-4, anti-PD-1 or combined (anti-CTLA-4 + anti-PD-1) treatment and compared with those of the untreated controls (Supplementary Table S2). The results of FLIM were then compared with the standard methods of response evaluation.

**Figure 6:**
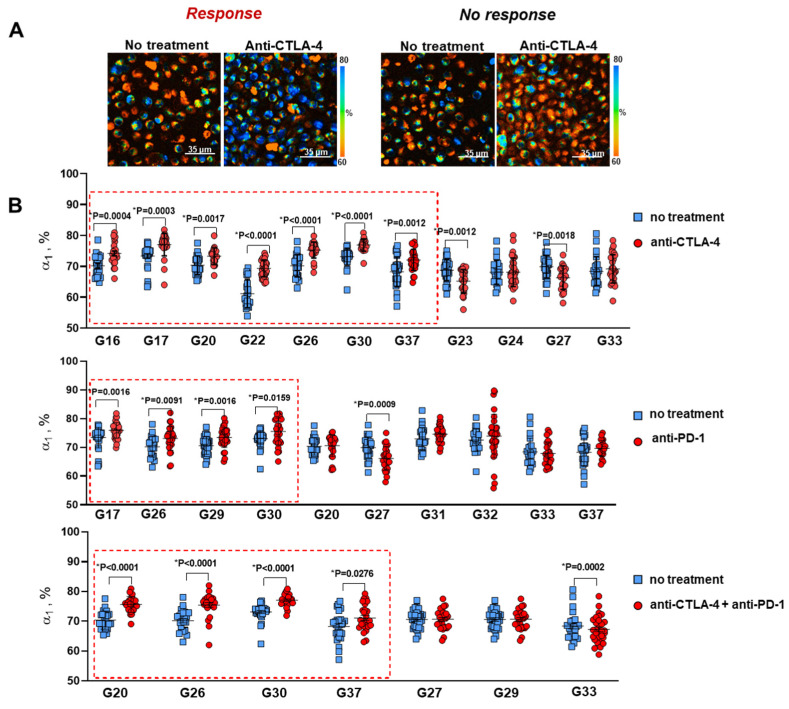
FLIM of NAD(P)H in lymphocytes from the G-EXP-L models after anti-CTLA-4, anti-PD-1 or combined (anti-CTLA-4 + anti-PD-1) treatment. (**A**) Representative FLIM images of lymphocytes from responding (patient G30) and non-responding (patient G27) models. The relative amplitude of free NAD(P)H α_1_ is shown in the untreated and treated T cells. Scale bar is indicated on the images. (**B**) Quantification of NAD(P)H α_1_ for individual patient-derived models. The graphs display the mean and SD (horizontal lines). Dots are the measurements for individual cells. The red boxes indicate the cases of a significant rise in NAD(P)H α_1_. Statistics: Student’s *t*-test. * Significant difference, *p* ≤ 0.05.

After the anti-CTLA-4 treatment, all “responders” (G16, G17, G20, G26, G30, and G37) and “partial responders” (G22) models showed a statistically significant increase in free NAD(P)H fraction α_1_ in lymphocytes in comparison with untreated samples (Figure 6). In four “non-responders” tested, α_1_ either did not change (G24, G33) or decreased (G24 and G27, *p* ≤ 0.0012).

After the anti-PD-1 treatment, “responder” G17, G26, G30, and “partial responder” G29 showed a statistical increase in α_1_ (for example, 72.1 ± 0.6 versus 70.1 ± 0.5%, *p* = 0.0016 and 74.7 ± 0.7 versus 73.1 ± 0.5%, *p* = 0.0159 for G30 and G29, respectively). For the “partial responder” G20 and most “non-responders” (G31, G32, G33, and G37) α_1_ did not change. For “non-responder” G27, a significant decrease in α_1_ (66.4 ± 0.7 versus 69.9 ± 0.8, *p* = 0.0009) was observed.

After the combined treatment, a significant increase in α1 was detected in all “responders” (G20, G26, G30, and G37). The “non-responsive” models G29 and G33 demonstrated no changes in a1, while G27 showed a decrease in α1 (66.8 ± 0.7 versus 69.9 ± 0.7%, *p* = 0.0018).

Therefore, an increase in NAD(P)H-α_1_ in lymphocytes was observed in all morphological “responders” (13 of 28 cases) and the absence of the changes or a decrease in α_1_—in all “non-responders” (12 of 28 cases) irrespective of the type of therapy. Morphological “partial responders” showed an increase of α_1_ only if the proliferation index Ki67 decreased (2 of 3 cases); otherwise, α_1_ did not change.

Fluorescence lifetime of protein-bound NAD(P)H τ_2_ increased as a result of immunotherapy in 10 of 13 cases of complete response (4 with anti-CTLA-4, 2 with anti-PD-1, and 4 with combined treatment) (Figure S3) and did not change in the “partial responders”. In the “non-responders”, τ_2_ was unchanged or decreased compared with control lymphocytes from untreated models.

The mean lifetime τ_m_ statistically decreased in only two cases—G22 after anti-CTLA-4 treatment and G17 after anti-PD-1 treatment (Supplementary Table S2). In all other cases, no change in τ_m_ was observed because the increase or decrease in the contribution of the free NAD(P)H fraction α_1_ was usually compensated by, respectively, an elongation or shortening of the lifetime of the bound NAD(P)H fraction τ_2_.

Therefore, FLIM of the autofluorescence of a patient’s lymphocytes demonstrated the sensitivity of the NAD(P)H lifetime to the treatment with immune checkpoint inhibitors, resolving the heterogeneous response of models from different patients to the different kinds of treatment. Of the decay parameters, a contribution of free NAD(P)H a1 is best correlated with other response assays and, consequently, can be considered as a reliable indicator of tumor-reactivity of T-cells caused by immunotherapy.

The responses of G-EXP-L models to anti-CTLA-4, anti-PD-1 treatment or their combinations assessed by different methods are summarized in Table 2.

## 4. Discussion

Immunotherapy using the inhibitors of the immune checkpoints such as anti-CTLA4 and anti-PD-1/PD-L1 antibodies led to increased overall survival of patients with different types of neoplastic tumors. However, only a part of patients responds to the therapy, while others do not respond or relapse after an initial response. Mechanisms of immunotherapy drug resistance can differ from patient to patient and be associated with the immunosuppressive tumor microenvironment or specifics of tumor cells [5]. The microenvironmental factors include different factors that have the immunosuppressive effect, e.g., hypoxia, acidic environment, dysfunctional blood vessels, dense stroma, tumor-associated fibroblasts, tumor-associated macrophages, chemokines, metabolites, etc. The intrinsic tumor factors include the loss of tumor antigen, abnormalities in antigen presentation and processing, alterations in signaling pathways, expression or exocytosis of immune checkpoint proteins, secretion of certain metabolites, etc. As such, personalized approaches are in high demand in the field of immunotherapy [28,29].

This study demonstrates that the selection of effective drugs for individual patients can be achieved through the utilization of a patient-specific in vitro system comprising fresh tumor explants and autologous peripheral lymphocytes, in conjunction with fluorescence lifetime imaging microscopy (FLIM) for the assessment of cellular responses.

In vitro models for personalization of immunotherapy are increasingly developing. The most popular approaches for creating a 3D tumor model include generation of organoids from the tumor stem and progenitor cells, the use of resected tumor tissue (e.g., explant cultures, tumoroid systems, tumor slices) and 3D-bioprinting technologies employing biological specimens (e.g., cells, proteins, or DNA) [8]. The immune microenvironment can be simulated by the presence of tumor infiltrating immune cells, exogenous immune cells from a patient or a healthy donor, immune cell lineages or CAR-T cells. Each of the models has its own advantages and disadvantages. For our study, we opted to utilize the tumor explant culture as a model for drug screening for the following reasons: (1) it is based on patient’s material; (2) it preserves the key genotypic and morphological characteristics of the original tumor and its microenvironment, including intra- and intertumor heterogeneity, immune, and stromal components; (3) it can be easily and inexpensively reproduced for a wide range of patients; (4) it provides a reliable response within a relatively short period of time. To resemble the recruitment of the immune cells into immunotherapy, the exogenous autologic peripheral T cells were added to the explant culture. Recent studies indicate that the T cell response to immune checkpoint inhibitors largely relies on peripheral T cells [30]. In the study by Shekarian et al., patients’ glioblastoma explant-based model was used to identify biomarkers of response to anti-CD47, anti–PD-1, or their combination in secretome of the explants, which required culturing in a perfusion bioreactor combined with highly multiplexed microscopy, soluble protein arrays, and mass spectrometry [19].

Co-culturing of the tumor explants and the T cells resulted in the activation of T cells, modification of their metabolic profile, and a certain degree of cytotoxicity, as evidenced by a reduction in the proliferation of glioma cells. These results are consistent with other studies showing the destruction of cancer cells in tumor explants during co-cultivation with immune cells from the peripheral blood of healthy donors or autologous patients [23,24]. However, the cytotoxic effect of immune cells on glioma cells was insufficient to destroy the explant structure.

Although immunotherapy by immune checkpoint inhibitors is not used in the clinic for gliomas, its potential efficacy against this type of tumor remains a topic of active investigation. There are several completed clinical trials testing immune checkpoint inhibitors for glioblastoma treatment. Satisfactory results were shown for the addition of neoadjuvant therapy by anti-PD-1 antibody pembrolizumab to the sustained adjuvant therapy after surgery which improved overall and progression-free survival [31]. However, monotherapy by anti-PD-1 antibody nivolumab as well as adding nivolumab to radiotherapy and temozolomide did not improve the survival of patients [32]. Nevertheless, nivolumab has been found to increase the expression of chemokines, the infiltration of immune cells, and the clonal diversity of T cell receptors in the tumor microenvironment [33]. Some other immune checkpoints, including CTLA-4, CD47, CD73, TIGIT, and CD137 were also studied in either clinical trials or preclinical research for gliomas [32]. It is known that tumor-infiltrating lymphocytes in glioma highly express a number of immune checkpoints as a result of T cell exhaustion. Therefore, exploration of the combined therapies and personalization approaches to increase the effectiveness of immune checkpoint inhibitors for gliomas is relevant.

Once immunotherapy with checkpoint inhibitors is approved for the treatment of glioma, the proposed personalized drug screen approach can be considered for patient stratification and treatment planning. In the meantime, the patient-specific platform developed can be adopted and applied to different tumor types for which immune checkpoint inhibitors are already in use. In addition, since T lymphocytes are the major players in eradicating tumor cells in various types of immunotherapies, the use of the proposed approach is not limited to checkpoint inhibitors, but can be extended to, for example, cytokines, CAR T cell therapy, T cell-based vaccines.

Alterations in the metabolic status of T cells accompany all cellular events associated with their tumor-reactivity (e.g., changes in expression profile, proliferation, etc.) upon immunotherapy, and thus, can potentially serve as a reliable predictive biomarker, which is sensitive to early immunological rearrangements [34,35]. PD-1 and CTLA-4 receptors have been found to reprogram T cell metabolism by suppressing glycolytic pathways and promoting lipolysis and fatty acid oxidation that impair T cell activation [36]. The immune checkpoint blockade is capable of rescuing exhausted T cells by enhancing their glucose influx and glycolysis via mTOR signaling and Myc induction, which allows IFN-γ production and improves their effector anti-tumor function [37]. FLIM of NAD(P)H and flavin coenzymes is a powerful tool to characterize metabolic status of T cells in their native state; however, the potential of this technique in the context of immunotherapy is largely unexplored. We have previously demonstrated the ability of FLIM to assess the immune response to tumor development [20] and to the anti-CTLA-4 therapy [38] by the measurements of the fluorescence lifetime of NAD(P)H in fresh lymphoid tissue. Meanwhile, FLIM of NAD(P)H has proven to be a promising approach to evaluating chemotherapy drug sensitivity of patient-derived glioma cells in explant cultures [39] and cell monolayers [18]. In the current study, using a patient-derived G-EXP-L model, we showed that changes in the autofluorescence decay profile reflect the degree of anti-tumor activity of lymphocytes under immune checkpoints treatment and can serve as a marker of drug sensitivity.

Our study has several limitations that are common in work on patient-derived models in vitro. Firstly, the number of drug tests that can be performed for a specific patient depends on the amount of tumor tissue available during surgical resection and on the quality of the material. In our study, only 6 of 14 patients had the three regimens tested (anti-CTLA-4, anti-PD-1, or combination), while others were limited to the number of viable cells in the explant culture. The number of patients included in the study is relatively small, which is reflected in the preliminary nature of the obtained data. Our intention is to pursue the research further with a larger cohort of patients. Secondly, although FLIM enables the monitoring of metabolism of live cells starting from the early stages of cultivation, the impact of the therapy on lymphocytes in G-EXP-L models was assessed in only the endpoint (day 14) in order to correlate FLIM parameters with the standard assays of cell response. Further investigation will be conducted into the early effects of immunotherapy of T-cell metabolism with the objective of optimizing the procedure and providing the results of the drug screening as early as possible. The final, and most significant, limitation is the inability to correlate with clinical outcomes, as immune checkpoint inhibitors are not currently used in the clinical protocols for glioma treatment. It is therefore not yet possible to reach a conclusion as to whether the proposed platform is capable of predicting the sensitivities of individual patients’ tumors to immunotherapy drugs. Nevertheless, the consistency of metabolic alterations in lymphocytes in response to therapeutic interventions with their activation and glioma explant morphology and proliferation substantiates the efficacy of the screening approach.

Plans for the future studies will include the optimization of the protocol of in vitro analysis (particularly in terms of investigating early and late effects on T cells), collecting data from a larger number of patients, and validating the platform in clinical trials. As immunotherapy can be associated with side effects, future trials also should include an assessment of treatment safety and side effects to fully evaluate the pros and cons of the treatment.

Prior to the implementation in clinical practice, the validity of the predictive test should be assessed in clinical trials, where the response of the in vitro model is compared with the real response of the same patient. Ideally, such tests should be performed on tumor biopsy material and should not exceed the time required for obtaining results from standard histopathology or molecular assays.

Current clinical standards for immunotherapy prescription either do not include any stratification of the patients with a specific tumor type (e.g., melanoma) or suggest patient selection based on PD-L1 expression by tumor cells (e.g., lung cancer) [40]. Therefore, metabolic alterations in T lymphocytes identified by FLIM in a patient-specific in vitro system could potentially serve as a predictive biomarker in selecting patients who will benefit from immunotherapy.

## 5. Conclusions

In the era of personalized medicine, it is crucial to identify individual patients who will respond to immunotherapies with a high probability in order to screen for different treatment options for potential non-responders. Drug screening on patient-specific models in vitro is considered a powerful approach to the prediction of therapy outcomes in patients and to preclinical testing of drug candidates. In this area, the choice of the model that would preserve the genotypic and phenotypic characteristics of the original tumor and the features of tumor microenvironment and the choice of the reliable method of response assessment remain a challenge. Here, we validated the model of tumor explant culture generated from patients’ glioma, enriched by autologous lymphocytes, to evaluate the potential efficacy of immune checkpoint inhibitors. The results of the study demonstrated that the metabolic profile of the lymphocytes co-cultured with tumor cells is sensitive to their tumor-reactivity and can serve as a marker of drug sensitivity. Our results also confirm the potential of using FLIM as a tool for the assessment of the metabolic state of lymphocytes in patient-specific in vitro models. We believe that the developed platform, based on tumor explants, lymphocytes, and FLIM, can help not only in patient selection, but also in the adjustment of the treatment schedule, and that it can be applied to different tumor types and different immunotherapy drugs. Plans for future studies will include the optimization of the protocol of in vitro analysis (particularly in terms of investigating early and late effects on T cells), collecting data from a larger number of patients, and validating the platform in clinical trials.

## Figures and Tables

**Table 1:** Clinicopathological data of the patients.

Sample Code	Age	Sex	Diagnosis	Grade	IDH- Status	Primary/ Recurrent	Treatment Before Surgery
G16	34	M	Oligoastrocytoma	3	Mutant	Primary	No
G17	46	F	Glioblastoma	4	Wild-type	Primary	No
G20	54	F	Glioblastoma	4	Wild-type	Recurrent	SR+RT+ TMZ
G22	40	F	Astrocytoma	3	Mutant	Primary	No
G23	72	F	Glioblastoma	4	Wild-type	Primary	No
G24	64	M	Glioblastoma	4	Wild-type	Primary	No
G26	50	F	Glioblastoma	4	Wild-type	Primary	No
G27	73	F	Oligodendroglioma	3	Mutant	Primary	No
G29	72	F	Glioblastoma	4	Wild-type	Primary	No
G30	49	M	Astrocytoma	4	Mutant	Recurrent	SR
G31	32	M	Astrocytoma	2	Mutant	Primary	No
G32	67	M	Astrocytoma	4	Mutant	Primary	No
G33	37	M	Astrocytoma	2	Mutant	Primary	No
G37	28	F	Oligodendroglioma	3	Mutant	Primary	No

“M” or “F” indicates male or female respectively; “IDH” indicates isocitrate dehydrogenase; “SR”, “RT” and “TMZ” indicate surgical resection, radiotherapy and temozolomide respectively.

**Table 2:** Summary of the effects of immunotherapy on the G-EXP-L models.

Sample Code	Type of Treatment	Light Microscopy	Flow Cytometry			FLIM	
		Morphological Response	↑ CD8+CD69+	↑ CD4+CD69+	↓ Ki67+ Glioma Cells	↑ α_1_	↑ τ_2_
G16	anti-CTLA-4						
G17	anti-CTLA-4						
	anti-PD-1						
G20	anti-CTLA-4						
	anti-PD-1						
	combination						
G22	anti-CTLA-4						
G23	anti-CTLA-4						
G24	anti-CTLA-4						
G26	anti-CTLA-4						
	anti-PD-1						
	combination						
G27	anti-CTLA-4						
	anti-PD-1						
	combination						
G29	anti-PD-1						
	combination						
G30	anti-CTLA-4						
	anti-PD-1						
	combination						
G31	anti-PD-1						
G32	anti-PD-1						
G33	anti-CTLA-4						
	anti-PD-1						
	combination						
G37	anti-CTLA-4						
	anti-PD-1						
	combination						

Red squares indicate complete response, pink squares—partial response, white squares—no response. The arrow ↓ or ↑ indicate, respectively, the decrease or increase in the appropriate parameter.

## Data Availability

Data are contained within the article.

## Supplementary Materials

The following supporting information can be downloaded at: https://www.mdpi.com/article/10.3390/cells14020097/s1, Table S1. The characteristics of patient-derived glioma cell cultures. Table S2. FLIM parameters of NAD(P)H in lymphocytes from the G-EXP-L models after anti-CTLA-4, anti-PD-1 or combined (anti-CTLA-4 + anti-PD-1) treatment. Figure S1. Phase contrast microscopy of patient-derived glioma explant, Day 6. Figure S2. Quantification of NAD(P)H α_1_, τ_2_ or τ_m_ in lymphocytes of a healthy donor after incubation with 3 BP (15 μM) [41] during 24 h or with rotenone (1 μM) [42] during 1 h. The graphs display the mean and SD (horizontal lines). Dots are the measurements for individual cells. Statistics: Student’s *t*-test. Figure S3. FLIM of NAD(P)H in lymphocytes from the G-EXP-L models after anti-CTLA-4, anti-PD-1 or combined (anti-CTLA-4 + anti-PD-1) treatment.

## References

[1] Muthukutty P., Woo H.Y., Ragothaman M., Yoo S.Y. (2023). Recent Advances in Cancer Immunotherapy Delivery Modalities. Pharmaceutics.

[2] Liu C., Yang M., Zhang D., Chen M., Zhu D. (2022). Clinical Cancer Immunotherapy: Current Progress and Prospects. Front. Immunol..

[3] Wojtukiewicz M.Z., Rek M.M., Karpowicz K., Górska M., Polityńska B., Wojtukiewicz A.M., Moniuszko M., Radziwon P., Tucker S.C., Honn K.V. (2021). Inhibitors of Immune Checkpoints-PD-1, PD-L1, CTLA-4-New Opportunities for Cancer Patients and a New Challenge for Internists and General Practitioners. Cancer Metastasis Rev..

[4] Chae Y.K., Arya A., Iams W., Cruz M.R., Chandra S., Choi J., Giles F. (2018). Current Landscape and Future of Dual Anti-CTLA4 and PD-1/PD-L1 Blockade Immunotherapy in Cancer; Lessons Learned from Clinical Trials with Melanoma and Non-Small Cell Lung Cancer (NSCLC). J. Immunother. Cancer.

[5] Said S.S., Ibrahim W.N. (2023). Cancer Resistance to Immunotherapy: Comprehensive Insights with Future Perspectives. Pharmaceutics.

[6] Wang D.-R., Wu X.-L., Sun Y.-L. (2022). Therapeutic Targets and Biomarkers of Tumor Immunotherapy: Response versus Non-Response. Signal Transduct. Target. Ther..

[7] Sankar K., Ye J.C., Li Z., Zheng L., Song W., Hu-Lieskovan S. (2022). The Role of Biomarkers in Personalized Immunotherapy. Biomark. Res..

[8] Shindo Y., Hazama S., Tsunedomi R., Suzuki N., Nagano H. (2019). Novel Biomarkers for Personalized Cancer Immunotherapy. Cancers.

[9] Shelton S.E., Nguyen H.T., Barbie D.A., Kamm R.D. (2021). Engineering Approaches for Studying Immune-Tumor Cell Interactions and Immunotherapy. iScience.

[10] Boucherit N., Gorvel L., Olive D. (2020). 3D Tumor Models and Their Use for the Testing of Immunotherapies. Front. Immunol..

[11] Mu P., Zhou S., Lv T., Xia F., Shen L., Wan J., Wang Y., Zhang H., Cai S., Peng J. (2023). Newly Developed 3D in Vitro Models to Study Tumor-Immune Interaction. J. Exp. Clin. Cancer Res..

[12] Rahman M.M., Wells G., Rantala J.K., Helleday T., Muthana M., Danson S.J. (2024). In-Vitro Assays for Immuno-Oncology Drug Efficacy Assessment and Screening for Personalized Cancer Therapy: Scopes and Challenges. Expert Rev. Clin. Immunol..

[13] Paillon N., Ung T.P.L., Dogniaux S., Stringari C., Hivroz C. (2024). Label-Free Single-Cell Live Imaging Reveals Fast Metabolic Switch in T Lymphocytes. Mol. Biol. Cell.

[14] Lakowicz J.R., Szmacinski H., Nowaczyk K., Johnson M.L. (1992). Fluorescence Lifetime Imaging of Free and Protein-Bound NADH. Proc. Natl. Acad. Sci. USA.

[15] Kolenc O.I., Quinn K.P. (2019). Evaluating Cell Metabolism Through Autofluorescence Imaging of NAD(P)H and FAD. Antioxid. Redox Signal.

[16] Datta R., Gillette A., Stefely M., Skala M.C. (2021). Recent Innovations in Fluorescence Lifetime Imaging Microscopy for Biology and Medicine. J. Biomed. Opt..

[17] Powley I.R., Patel M., Miles G., Pringle H., Howells L., Thomas A., Kettleborough C., Bryans J., Hammonds T., MacFarlane M. (2020). Patient-Derived Explants (PDEs) as a Powerful Preclinical Platform for Anti-Cancer Drug and Biomarker Discovery. Br. J. Cancer.

[18] Yuzhakova D.V., Sachkova D.A., Shirmanova M.V., Mozherov A.M., Izosimova A.V., Zolotova A.S., Yashin K.S. (2023). Measurement of Patient-Derived Glioblastoma Cell Response to Temozolomide Using Fluorescence Lifetime Imaging of NAD(P)H. Pharmaceuticals.

[19] Shekarian T., Zinner C.P., Bartoszek E.M., Duchemin W., Wachnowicz A.T., Hogan S., Etter M.M., Flammer J., Paganetti C., Martins T.A. (2022). Immunotherapy of Glioblastoma Explants Induces Interferon-γ Responses and Spatial Immune Cell Rearrangements in Tumor Center, but Not Periphery. Sci. Adv..

[20] Izosimova A.V., Shirmanova M.V., Shcheslavskiy V.I., Sachkova D.A., Mozherov A.M., Sharonov G.V., Zagaynova E.V., Yuzhakova D.V. (2022). FLIM of NAD(P)H in Lymphatic Nodes Resolves T-Cell Immune Response to the Tumor. Int. J. Mol. Sci..

[21] German Y., Vulliard L., Kamnev A., Pfajfer L., Huemer J., Mautner A.-K., Rubio A., Kalinichenko A., Boztug K., Ferrand A. (2021). Morphological Profiling of Human T and NK Lymphocytes by High-Content Cell Imaging. Cell Rep..

[22] Lin W., Suo Y., Deng Y., Fan Z., Zheng Y., Wei X., Chu Y. (2015). Morphological Change of CD4^+^ T Cell during Contact with DC Modulates T-Cell Activation by Accumulation of F-Actin in the Immunology Synapse. BMC Immunol..

[23] M Kholosy W., Derieppe M., van den Ham F., Ober K., Su Y., Custers L., Schild L., M J van Zogchel L., M Wellens L., R Ariese H. (2021). Neuroblastoma and DIPG Organoid Coculture System for Personalized Assessment of Novel Anticancer Immunotherapies. J. Pers. Med..

[24] Dijkstra K.K., Cattaneo C.M., Weeber F., Chalabi M., van de Haar J., Fanchi L.F., Slagter M., van der Velden D.L., Kaing S., Kelderman S. (2018). Generation of Tumor-Reactive T Cells by Co-Culture of Peripheral Blood Lymphocytes and Tumor Organoids. Cell.

[25] Hu C., Xuan Y., Zhang X., Liu Y., Yang S., Yang K. (2022). Immune Cell Metabolism and Metabolic Reprogramming. Mol. Biol. Rep..

[26] Leone R.D., Powell J.D. (2020). Metabolism of Immune Cells in Cancer. Nat. Rev. Cancer.

[27] Seidel J.A., Otsuka A., Kabashima K. (2018). Anti-PD-1 and Anti-CTLA-4 Therapies in Cancer: Mechanisms of Action, Efficacy, and Limitations. Front. Oncol..

[28] Bai R., Chen N., Li L., Du N., Bai L., Lv Z., Tian H., Cui J. (2020). Mechanisms of Cancer Resistance to Immunotherapy. Front. Oncol..

[29] Piper M., Kluger H., Ruppin E., Hu-Lieskovan S. (2023). Immune Resistance Mechanisms and the Road to Personalized Immunotherapy. Am. Soc. Clin. Oncol. Educ. Book.

[30] Yost K.E., Chang H.Y., Satpathy A.T. (2021). Recruiting T Cells in Cancer Immunotherapy. Science.

[31] Cloughesy T.F., Mochizuki A.Y., Orpilla J.R., Hugo W., Lee A.H., Davidson T.B., Wang A.C., Ellingson B.M., Rytlewski J.A., Sanders C.M. (2019). Neoadjuvant Anti-PD-1 Immunotherapy Promotes a Survival Benefit with Intratumoral and Systemic Immune Responses in Recurrent Glioblastoma. Nat. Med..

[32] Yasinjan F., Xing Y., Geng H., Guo R., Yang L., Liu Z., Wang H. (2023). Immunotherapy: A Promising Approach for Glioma Treatment. Front. Immunol..

[33] Schalper K.A., Rodriguez-Ruiz M.E., Diez-Valle R., López-Janeiro A., Porciuncula A., Idoate M.A., Inogés S., de Andrea C., López-Diaz de Cerio A., Tejada S. (2019). Neoadjuvant Nivolumab Modifies the Tumor Immune Microenvironment in Resectable Glioblastoma. Nat. Med..

[34] Ma S., Ming Y., Wu J., Cui G. (2024). Cellular Metabolism Regulates the Differentiation and Function of T-Cell Subsets. Cell Mol. Immunol..

[35] Aksoylar H.-I., Tijaro-Ovalle N.M., Boussiotis V.A., Patsoukis N. (2020). T Cell Metabolism in Cancer Immunotherapy. Immunometabolism.

[36] Patsoukis N., Bardhan K., Chatterjee P., Sari D., Liu B., Bell L.N., Karoly E.D., Freeman G.J., Petkova V., Seth P. (2015). PD-1 Alters T-Cell Metabolic Reprogramming by Inhibiting Glycolysis and Promoting Lipolysis and Fatty Acid Oxidation. Nat. Commun..

[37] Chang C.-H., Qiu J., O’Sullivan D., Buck M.D., Noguchi T., Curtis J.D., Chen Q., Gindin M., Gubin M.M., van der Windt G.J.W. (2015). Metabolic Competition in the Tumor Microenvironment Is a Driver of Cancer Progression. Cell.

[38] Yuzhakova D.V., Lukina M.M., Sachkova D.A., Yusubalieva G.M., Baklaushev V.P., Mozherov A.M., Dudenkova V.V., Gavrina A.I., Yashin K.S., Shirmanova M.V. (2023). Development of a 3D Tumor Spheroid Model from the Patient’s Glioblastoma Cells and Its Study by Metabolic Fluorescence Lifetime Imaging. Sovrem. Tekhnologii Med..

[39] Morelli M., Lessi F., Barachini S., Liotti R., Montemurro N., Perrini P., Santonocito O.S., Gambacciani C., Snuderl M., Pieri F. (2022). Metabolic-Imaging of Human Glioblastoma Live Tumors: A New Precision-Medicine Approach to Predict Tumor Treatment Response Early. Front. Oncol..

[40] Kovács S.A., Fekete J.T., Győrffy B. (2023). Predictive Biomarkers of Immunotherapy Response with Pharmacological Applications in Solid Tumors. Acta Pharmacol. Sin..

[41] Roy S., Dukic T., Bhandary B., Tu K.J., Molitoris J., Ko Y.H., Shukla H.D. (2022). 3-Bromopyruvate Inhibits Pancreatic Tumor Growth by Stalling Glycolysis, and Dismantling Mitochondria in a Syngeneic Mouse Model. Am. J. Cancer Res..

[42] Walsh A.J., Mueller K.P., Tweed K., Jones I., Walsh C.M., Piscopo N.J., Niemi N.M., Pagliarini D.J., Saha K., Skala M.C. (2020). Classification of T-Cell Activation via Autofluorescence Lifetime Imaging. Nat. Biomed. Eng..

