# Supplementary material for: Fluorescence Lifetime Imaging of NAD(P)H in Patients’ Lymphocytes: Evaluation of Efficacy of Immunotherapy
Source: Cells. 2025 Jan 10;14(2):97. doi: 10.3390/cells14020097 (PMC11764258; doi:10.3390/cells14020097)
Supplement: Supplementary file 1 [file cells-14-00097-s001.zip › cells-3343521-supplementary.pdf]

1. Supplementary Materials and Methods

1.1 Patient-Derived Explants of Glioma

The fresh tumor specimen was washed in DMEM/F12 with 2% antibiotic-antimycotic and dissected with a scalpel into fragments of approximately 500 μm^3, which were plated in 12-well plates (4-5 fragments per well) and incubated in a CO_2 incubator (37°C, 5% CO_2 and 85% humidity) in RPMI-1640 medium with L-glutamine and HEPES (Gibco, Amaril-lo, TX, USA) with addition of 10% fetal bovine serum (FBS) and 1% antibiotic–antimycotic (Gibco, Amarillo, TX, USA). Depending on the amount of resection material, the total number of wells for each patient varied from 5 to 12.

After 72-h incubation, the cells began to detach from the explant, migrate radially and form a distinctive "sun shape" structure. The culture medium was replaced every 72 h, and any debris was removed.

1.2. Lymphocyte Isolation and Cultivation

The whole blood was carefully layered onto Histopaque-1077, centrifuged at 400× *g* for 30 min at room temperature. The opaque interface containing mononuclear cells was carefully collected and washed with phosphate-buffered saline (PBS). The erythrocytes were lysed by adding ACK Lysing Buffer (Gibco, Amarillo, TX, USA) (1:15) for 5 min, then the cells were washed with PBS by centrifuging at 300× *g* for 5 min.

The resulting immune cells were cultured in RPMI-1640 medium with L-glutamine and HEPES with addition of 0.1% human interleukin 2, 10% fetal bovine serum and 0.1% penicillin-streptomycin on 25-cm^2 culture flasks in a CO_2 incubator at 37°C, 5% CO_2 and 85% humidity. Sub-cultivation was performed once or twice a week with centrifugation at 300× *g* for 5 min.

1.3. FLIM of NAD(P)H in Lymphocytes.

FLIM of NAD(P)H was carried out using an LSM 880 laser scanning confocal microscope (Carl Zeiss, Germany) equipped with an FLIM module Simple Tau 152 TCSPC (Becker & Hickl GmbH, Berlin, Germany) and a hybrid detector HPM-100-40 (Becker and Hickl GmbH, Berlin, Germany). A femtosecond Ti:Sapphire laser (80 MHz, 140 fs) was used for two-photon excitation of NAD(P)H fluorescence at 750 nm. Fluorescence of NAD(P)H was detected at 450–490 nm by a combination of a 490LP dichroic mirror and a ET475/50 bandpass filter (Chroma, US). The average laser power at the samples was about 6 mW. A C Plan-Apochromat 40×/1.3 NA Oil DIC objective was used for image acquisition. The field of view was 213 × 213 μm (512 × 512 pixels). Acquisition time of the images was 60 s. During FLIM image acquisition, the cells were maintained in the stage top incubator at 37°C and 5% CO2.

FLIM images were processed in the SPCImage 8.3 software (Becker & Hickl GmbH, Germany). On average 5000-10000 photons were collected per decay curve at binning factor 3. Fitting was performed using a bi-exponential decay model using weighted least square algorithm. The goodness of the fit χ_2 was 0.8-1.2. The values of the short and long components of the lifetimes (τ_1 and τ_2) and their relative contributions (α_1 and α_2, $\alpha_1 + \alpha_2 = 100\%$) were obtained, which correspond to the free and protein-bound forms of the NAD(P)H cofactor, respectively. The weighted average (mean) lifetime was calculated as $\tau_m = (\alpha_1 * \tau_1 + \alpha_2 * \tau_2)/(\alpha_1 + \alpha_2)$. Fluorescence lifetimes were analyzed in cell cytoplasm by manual selection of the maximal area of cytoplasm as region of interest in each individual cell. For each sample, FLIM images were acquired from 2-3 fields of view, with a total number of cells used for calculations 30-40.

1.4. Flow Cytometry

For analysis by flow cytometry the immune cells after centrifugation were resuspended in 100 mL of in phosphate-buffered saline (PBS) and stained for CD3, CD8, and CD4 surface antigens to identify T-cell subsets, and for the expression of the cell surface markers of early (CD69) and middle (CD25 (IL-2Rα)) activation. The following antibodies were used: anti-human CD3-Allophycocyanin, CD8-Allophycocyanin-Cyanine-7, CD4-Brilliant violet 605, CD25-Phycoerythrin and CD69-Phycoerythrin-Cyanine-7 (BD Biosciences, San Jose, CA, USA).

To evaluate the proliferative index Ki67 of glioma cells, the tumor cells were removed from the plate, centrifuged at 200× *g* for 5 min and resuspended in PBS. The intracellular staining (ICS) was performed using the Inside Stain Kit (Miltenyi Biotec, Bergisch Gladbach, Germany) according to the manufacturer's protocol. The glioma cells were fixed, permeabilized and stained with an antibody Ki67-Brilliant violet 425 (BioLegend, San Diego, USA).

1.4. Metabolic inhibitors

Lymphocytes, isolated from the blood of a healthy donor and expanded as described above, were incubated with the metabolic inhibitor 3-bromopyruvate (3-BP) (15 μM) [41] for 24 h, or rotenone (1 μM) [42] for 1 h.

2. Supplementary Results

Supplementary Table S1. The characteristics of patient-derived glioma cell cultures.

Sample Code	GFAP*	Ki67, %	Cell polymorphism**
G16	+	-	+
G17	+	46 ± 1.2	+
G20	+	45 ± 1.5	+
G22	+	70 ± 2.9	+
G23	+	-	+
G24	+	-	++
G26	+	50 ± 3.2	++
G27	+	69 ± 2.1	++
G29	+	65 ± 1.2	+
G30	+	41 ± 1.3	+
G31	+	46 ± 2.5	++
G32	+	51 ± 3.2	++
G33	+	24 ± 2.6	+
G37	+	26 ± 1.9	+

*, + corresponds to GFAP-positive cultures; **, + or ++ indicate the degree of cell polymorphism.

Supplementary Table S2. FLIM parameters of NAD(P)H in lymphocytes from the G-EXP-L models after anti-CTLA-4, anti-PD-1 or combined (anti-CTLA-4 + anti-PD-1) treatment.

Sample code	Treatment		FLIM parameters of NAD(P)H				
			τ_m	τ_1	τ_2	α_1	α_2
G16	After co-culturing	no treatment	1.06±0.04	0.41±0.01	2.59±0.05	70.2±0.6	29.81±0.6
		anti-CTLA-4	0.96±0.04	0.38±0.02	2.62±0.05	74.2±0.5*	25.8±0.5*
G17	After co-culturing	no treatment	0.97±0.03	0.45±0.02	2.39±0.04	73.4±0.5	26.62±0.5
		anti-CTLA-4	0.95±0.03	0.46±0.01	2.61±0.04*	77.1±0.6*	22.96±0.6*
		anti-PD-1	0.87±0.04*	0.47±0.02	2.54±0.05*	75.9±0.5*	24.09±0.5*
G20	Before co-culturing		1.35±0.05	0.53±0.02	3.18±0.09	69.1±0.8	30.9±0.8
	After co-culturing	no treatment	0.98±0.02	0.37±0.01	2.45±0.04	70.4±0.6	29.6±0.6
		anti-CTLA-4	1.00±0.02	0.39±0.01	2.67±0.04*	73.3±0.5*	26.7±0.5*
		anti-PD-1	1.04±0.02	0.41±0.01	2.57±0.04	70.6±0.7	29.4±0.7
		combination	0.93±0.02	0.39±0.01	2.61±0.05*	75.6±0.5*	24.2±0.5*
G22	Before co-culturing		1.31±0.05	0.44±0.03	3.00±0.09	66.2±0.5	33.8±0.8
	After co-culturing	no treatment	1.27±0.04	0.47±0.02	2.55±0.05	67.1±0.6	32.9±0.6
		anti-CTLA-4	1.06±0.03*	0.38±0.01	2.59±0.05	69.7±0.5*	30.7±0.5*
G23	After co-culturing	no treatment	1.00±0.02	0.28±0.02	2.59±0.06	68.8±0.6	31.23±0.6
		anti-CTLA-4	1.03±0.02	0.32±0.03	2.35±0.06*	65.2±0.6	34.77±0.6
G24	After co-culturing	no treatment	1.06±0.02	0.34±0.02	2.59±0.07	68.1±0.7	31.88±0.7
		anti-CTLA-4	1.08±0.03	0.37±0.02	2.58±0.07	68.1±0.6	31.91±0.6
G26	After co-culturing	no treatment	0.99±0.03	0.39±0.03	2.42±0.03	70.2±0.5	29.82±0.5
		anti-CTLA-4	0.95±0.02	0.42±0.03	2.58±0.03*	75.3±0.7	24.74±0.7
		anti-PD-1	1.01±0.03	0.41±0.01	2.63±0.05*	73.1±0.5	26.91±0.5
		combination	0.97±0.04	0.43±0.03	2.62±0.05*	75.4±0.6	24.56±0.6

G27	Before co-culturing		1.34±0.05	0.45±0.02	3.08±0.09	66.2±0.8	33.8±0.8
	After co-culturing	no treatment	0.89±0.02	0.31±0.01	2.28±0.03	69.9±0.7	30.1±0.7
		anti-CTLA-4	0.90±0.03	0.32±0.01	2.06±0.03*	66.4±0.7*	33.6±0.7*
		anti-PD-1	0.90±0.03	0.28±0.01	2.12±0.03*	66.4±0.8*	33.6±0.8*
		combination	0.94±0.03	0.31±0.01	2.21±0.03	66.8±0.7*	33.2±0.7*
G29	Before co-culturing		1.29±0.04	0.49±0.03	2.94±0.02	68.2±0.7	32.4±0.7
	After co-culturing	no treatment	0.95±0.01	0.35±0.01	2.35±0.02	70.1±0.5	29.8±0.5
		anti-PD-1	0.96±0.01	0.40±0.01	2.41±0.05	72.1±0.6*	28.0±0.6*
		combination	0.93±0.03	0.29±0.01	2.41±0.04	68.7±0.7	30.1±0.7
G30	Before co-culturing		1.37±0.04	0.62±0.02	3.2±0.07	70.9±1.3	29±1.3
	After co-culturing	no treatment	0.88±0.01	0.35±0.01	2.34±0.03	73.1±0.5	26.9±0.5
		anti-CTLA-4	0.89±0.01	0.39±0.01	2.50±0.04*	76.9±0.4*	23.1±0.4*
		anti-PD-1	0.87±0.03	0.35±0.01	2.41±0.04	74.7±0.7*	25.3±0.7*
		combination	0.87±0.01	0.37±0.01	2.56±0.04*	77.3±0.2*	22.7±0.2*
G31	Before co-culturing		1.30±0.04	0.52±0.01	3.15±0.05	70.3±0.6	29.7±0.6
	After co-culturing	no treatment	0.93±0.02	0.45±0.01	2.40±0.06	72.9±0.8	27.1±0.8
		anti-PD-1	0.91±0.02	0.41±0.01	2.45±0.07	74.6±0.7	25.4±0.7
G32	Before co-culturing		1.24±0.04	0.46±0.01	3.05±0.06	69.8±1.1	30.2±1.1
	After co-culturing	no treatment	0.97±0.02	0.41±0.01	2.47±0.04	72.5±0.8	27.5±0.8
		anti-PD-1	0.97±0.03	0.43±0.01	2.51±0.04	72±1.2	27±1.2
G33	Before co-culturing		1.39±0.05	0.54±0.03	3.12±0.07	67.0±0.8	33.1±0.8
	After co-culturing	no treatment	1.22±0.03	0.45±0.02	2.92±0.07	68.3±0.9	31.7±0.9
		anti-CTLA-4	1.30±0.05	0.50±0.02	3.0±0.1	69.3±0.9	31.7±0.9
		anti-PD-1	1.12±0.02	0.42±0.02	2.62±0.05*	67.9±0.8	32.2±0.8
		combination	1.15±0.03	0.44±0.02	2.65±0.06*	67.3±0.9	32.7±0.9
G37	Before co-culturing		1.32±0.05	0.53±0.03	3.05±0.08	67.5±0.9	32.5±0.9
	After co-culturing	no treatment	1.12±0.02	0.41±0.01	2.66±0.06	68.2±0.4	31.8±0.4
		anti-CTLA-4	1.05±0.02	0.43±0.01	2.64±0.05	72.1±0.7*	27.9±0.7*
		anti-PD-1	1.08±0.02	0.40±0.01	2.69±0.04	70.1±0.8	29.9±0.8
		combination	1.14±0.01	0.42±0.01	2.77±0.04*	70.8±0.4*	30.29±0.4*

* statistically significant difference compared to the corresponding group "no treatment", $p \leq 0.05$. Data are presented as means ± SD.

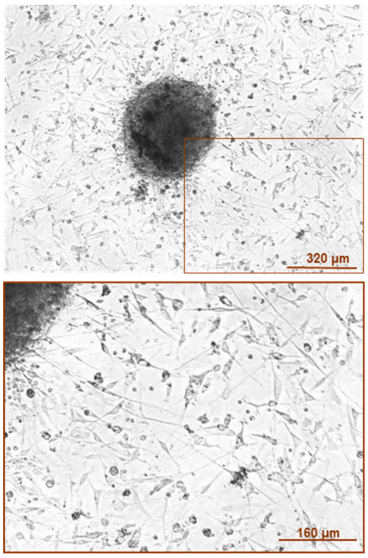

Supplementary Figure S1. Phase contrast microscopy of patient-derived glioma explant, Day 6.

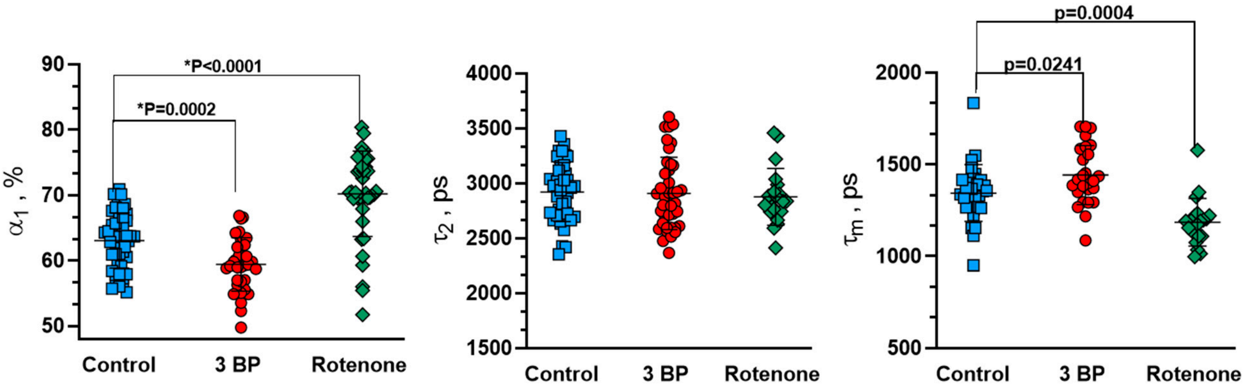

Supplementary Figure S2. Quantification of NAD(P)H α_1 , τ_2 or τ_m in lymphocytes of a healthy donor after incubation with 3 BP (15 μM) during 24 h or with rotenone (1 μM) during 1 h. The graphs display the mean and SD (horizontal lines). Dots are the measurements for individual cells. Statistics: Student's t-test. *Significant difference, $p \leq 0.05$.

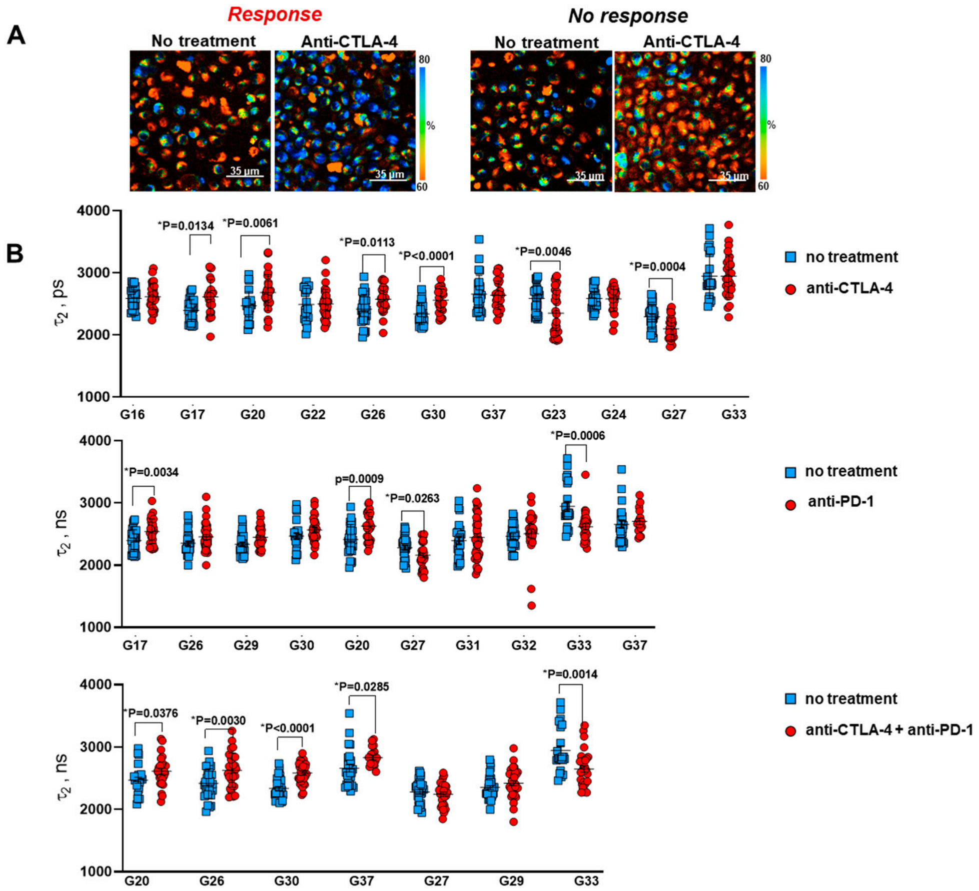

Supplementary Figure S3. FLIM of NAD(P)H in lymphocytes from the G-EXP-L models after anti-CTLA-4, anti-PD-1 or combined (anti-CTLA-4 + anti-PD-1) treatment. (A) Representative FLIM images of lymphocytes from responding (patient G30) and non-responding (patient G27) models. The lifetime of protein-bound NAD(P)H τ_2 is shown in the untreated and treated T cells. Scale bar is indicated on the images. (B) Quantification of NAD(P)H τ_2 for individual patient-derived models. The graphs display the mean and SD (horizontal lines). Dots are the measurements for individual cells. Statistics: Student's t-test. *Significant difference, $p \leq 0.05$.
